# Supplementary material for: Widespread use of ChatGPT and other Artificial Intelligence tools among medical students in Uganda: A cross-sectional study
Source: PLoS One. 2025 Jan 9;20(1):e0313776. doi: 10.1371/journal.pone.0313776 (PMC11717177; doi:10.1371/journal.pone.0313776)
Supplement: Attachment — Submitted filename: Rebuttal letter AI_070424.docx [file pone.0313776.s003.docx]

**Re: "Widespread use of ChatGPT and other Artificial Intelligence tools among medical students in Uganda: a cross-sectional study "**

Thank you for reviewing our manuscript.

Below is our point-by-point response to each of the comments.

Thank you!

| **Reviewer’s comment** | **Response to comment** | **Line number** |
| --- | --- | --- |
| Academic Editor | | |
| 1. Data visualization could be improved. Could we present some of the figures using charts other than bar graphs?. For bar charts, please indicate the axis titles. | Thank you for this comment, | N/A |
| 2. Please expand the DISCUSSION. There are new reports on this aspect in other countries, which may make your discussion robust, and probably lead to more meaningful conclusions. | Thank you, we have revised the discussion basing on findings from new articles on Artificial Intelligence in medical education. |  |
| 3. It is necessary to indicate the potential limitations of the study, just as you indicated the strengths. | Thank you, we have described the limitations of our study.  “*One key limitation of our study is the reliance on self-reported data from medical students, which might be subject to social desirability bias. However, to reduce social desirability bias, we used a self-administered questionnaire for data collection*” | 280-282 |
| 4. Your conclusions should be expanded in the broader context, and should preferably give some directions for future research. | Thank you, this has been done. | 284-290 |
| **Reviewer #1** | | |
| Title: The title could be improved removing redundant words by specifying the type of AI tools examined in the study. For example, "Widespread use of ChatGPT and Other Artificial Intelligence Tools among Medical Students in Uganda: A Cross-Sectional Study." | Thank you for your comment. However, the other AI tools used are many and shall make the title very lengthy. | N/A |
| Abstract: Consider including specific findings or percentages in the abstract to give readers a clearer understanding of the key results. Also, mentioning the implications of the findings in the abstract would enhance its significance. | Thank you, this has been done. | 40-55 |
| Introduction: The introduction effectively sets the context by explaining the significance of AI tools like ChatGPT in medical education. However, it could be strengthened by providing more context on the challenges faced by medical education due to the widespread adoption of AI tools. Consider integrating more recent literature or studies to emphasize the relevance and timeliness of the research. | Thank you, this has been done. | 69-88 |
| Materials and Methods: Provide more details on the semi-structured questionnaire used, including the specific questions related to AI tool usage. Describe the process of questionnaire adaptation and validation, if applicable, to ensure the reliability of data collection instruments. | Thank you, this has been done | 136-140 |
| Mention the steps taken to ensure the confidentiality and anonymity of participants' responses. | Thank you, this has been done | 298-301 |
| Discussion: The discussion effectively contextualizes the findings within existing literature and highlights their implications. However, the authors should provide a more detailed comparison of the study findings with similar research conducted in other settings or populations. | We have revised the discussion accordingly. | 206-271 |
| While the authors discuss the strength of the, they should consider discussing potential limitations of the study and their impact on the interpretation of results. Also, propose recommendations for future research or practical interventions based on the study findings. | Thank you, we have described the limitations of our study.  “One key limitation of our study is the reliance on self-reported data from medical students, which might be subject to social desirability bias. However, to reduce social desirability bias, we used a self-administered questionnaire for data collection” | 278-281 |
| Conclusion: Consider reiterating the implications of the study findings for medical education policy and practice. Provide clear recommendations for addressing the challenges and opportunities associated with the widespread use of AI tools among medical students. Avoid introducing new information or findings in the conclusion section. | Thank you, we have revised accordingly. | 283-289 |
| Ethical Approval and Consent to Participate: Consider including information on how participants were informed about the study objectives, risks, and benefits before obtaining their consent. Specify whether any incentives or compensation were provided to participants for their involvement in the study. | Thank you for the comment. This has been done. | 293-300 |
| General Comments: The article would benefit from thorough proofreading to correct grammatical errors, typos, and formatting inconsistencies. | Thank you, we have done a proofreading and corrected the identified grammatical errors, and typos | N/A |
| Reviewer #2 |  |  |
| 1. “In order to exploit the use of ChatGPT and other related AI software, there is an urgent need to develop guidelines.” – do the authors mean to say, in order to prevent exploitation? | Thank you for your comment. With exploiting, we refer to making use of. We have rephrased it to *“AI tools can address faculty shortages, support research, and innovation, and foster critical thinking skills. In order to fully exploit these benefits, there is an urgent need to develop institutional AI policies and guidelines, thereby harnessing the advantages while mitigating associated risks in medical education”* |  |
| 2. What were the measures taken to reduce social desirability bias in the study? Will students actually acknowledge its use for plagiarism? | To reduce social desirability bias, we used a self-administered questionnaire. | 136 |
| 3. Are there any guidelines in developed countries / HMICs on how to use AI tools? | Yes, there are guidelines in some high-income countries on AI use in education | N/A |
| 4. How can guidelines be formed to limit its misuse? – Will universities / journals check for AI written content? |  |  |
| 5. “Almost all 93% (522/564) had ever had about AI tools 155 such as ChatGPT.” – Needs to be rephrased | Thank you, this has been rephrased. | 162 |
| 6. “Most 71%” – most and percentage should be avoided together | Thank you, this has been rephrased. | 163 |
| 7. Results need to be rewritten in a better language | Thank you, this has been done. | 158-204 |
| 8. “Medical students aged 35 to 46 years were 31% less likely to use AI tools as compared to those aged less than 35 years” – Are undergraduate medical students aged above 35, this is highly unusual across the world? Also can 13 really be compared against 47 and 77 to draw such a definite conclusion? | We understand your concern. However, in Uganda, there are undergraduate medical students who are aged more than 35 years. These majorly include those who study medical course at diploma level and later join medical school for a medical undergraduate bachelor degree. | N/A |
| 9. While the study provides insights on the patterns on Generative AI use, and the need for regulatory guidelines – Can the authors discuss steps taken in other countries to reduce its misuse. | Thank you, we have discussed these as indicated. Other countries have developed AI policies and guidelines to prevent AI misuse. | 268-272 |
