# Supplementary material for: Widespread use of ChatGPT and other Artificial Intelligence tools among medical students in Uganda: A cross-sectional study
Source: PLoS One. 2025 Jan 9;20(1):e0313776. doi: 10.1371/journal.pone.0313776 (PMC11717177; doi:10.1371/journal.pone.0313776)
Supplement: Attachment — Submitted filename: Reviewer comments.pdf [file pone.0313776.s002.pdf]

I am pleased to give my thoughts on the article titled 'Widespread Use of ChatGPT and Other Artificial Intelligence Tools Among Medical Students in Uganda: A Cross-Sectional Study.' Overall, it appears to be a promising article of good quality. However, it can be improved.

Title: The title could be improved removing redundant words by specifying the type of AI tools examined in the study. For example, "Widespread use of ChatGPT and Other Artificial Intelligence Tools among Medical Students in Uganda: A Cross-Sectional Study."

Abstract: Consider including specific findings or percentages in the abstract to give readers a clearer understanding of the key results. Also, mentioning the implications of the findings in the abstract would enhance its significance.

Introduction: The introduction effectively sets the context by explaining the significance of AI tools like ChatGPT in medical education. However, it could be strengthened by providing more context on the challenges faced by medical education due to the widespread adoption of AI tools. Consider integrating more recent literature or studies to emphasize the relevance and timeliness of the research.

Materials and Methods: Provide more details on the semi-structured questionnaire used, including the specific questions related to AI tool usage. Describe the process of questionnaire adaptation and validation, if applicable, to ensure the reliability of data collection instruments.

Mention the steps taken to ensure the confidentiality and anonymity of participants' responses.

Results: The results section presents the key findings of the study in a structured manner.

Discussion: The discussion effectively contextualizes the findings within existing literature and highlights their implications. However, the authors should provide a more detailed comparison of the study findings with similar research conducted in other settings or populations.

While the authors discuss the strength of the, they should consider discussing potential limitations of the study and their impact on the interpretation of results. Also, propose recommendations for future research or practical interventions based on the study findings.

Conclusion: Consider reiterating the implications of the study findings for medical education policy and practice. Provide clear recommendations for addressing the challenges and opportunities associated with the widespread use of AI tools among medical students. Avoid introducing new information or findings in the conclusion section.

Ethical Approval and Consent to Participate: Consider including information on how participants were informed about the study objectives, risks, and benefits before obtaining their consent. Specify whether any incentives or compensation were provided to participants for their involvement in the study.

General Comments: The article would benefit from thorough proofreading to correct grammatical errors, typos, and formatting inconsistencies.
